# Prevalence of radiographic findings of femoroacetabular impingement in the Japanese population

**DOI:** 10.1186/1749-799X-9-25

**Published:** 2014-04-11

**Authors:** Kensuke Fukushima, Katsufumi Uchiyama, Naonobu Takahira, Mitsutoshi Moriya, Takeaki Yamamoto, Moritoshi Itoman, Masashi Takaso

**Affiliations:** 1 Department of Orthopaedic Surgery, Kitasato University School of Medicine, 1-15-1 Kitasato, Minami-ku, Sagamihara, Kanagawa 252-0374, Japan; 2 Department of Rehabilitation, Kitasato University School of Allied Health Sciences, 1-15-1 Kitasato, Minami-ku, Sagamihara, Kanagawa 252-0374, Japan; 3 Department of Orthopaedic Surgery, Kyusyuu Rosai Hospital, 1-3-1 Kuzuhara, Takamatsu, Kokura-minami-ku, Kitakyusyu, Fukuoka 800-0296, Japan

**Keywords:** Femoroacetabular impingement, Osteoarthritis of the hip, Acetabular retroversion, Hip dysplasia, Radiographic study

## Abstract

**Background:**

Femoroacetabular impingement (FAI) is one factor known to cause pain and osteoarthritis (OA) of the hips. Although secondary OA due to hip dysplasia is common among Japanese populations, primary OA is seldom observed. Concomitantly, FAI is also thought to be uncommon in Japan, but relatively few epidemiological studies have addressed this issue. This study aimed to clarify the prevalence of radiographic findings of FAI in a Japanese population and to evaluate whether FAI is a risk factor for the development of arthritic changes.

**Methods:**

We retrospectively examined 87 patients who underwent unilateral hip osteotomy with a Charnley category A hip joint on the contralateral side. Anteroposterior-view radiographs of the non-operated hip joint were assessed for the presence of hip dysplasia, as well as pistol grip deformity and crossover sign indicative of cam-type and pincer-type impingement, respectively. The presence of arthritic changes in the non-operated hip joint was assessed in follow-up radiographs, and factors contributing to the development of arthritis were determined by survival analysis.

**Results:**

Of the 87 hips examined, dysplasia was noted in 38 (43.6%). While no pistol grip deformity was observed, crossover sign, which is indicative of pincer-type impingement, was identified in 9 of 38 dysplastic hips (23.7%) and 15 of 49 non-dysplastic hips (30.6%). Arthritic changes were present in 13 of 38 dysplastic hips (34.2%) and 11 of 49 non-dysplastic hips (22.4%). Survival analysis revealed that the presence of the crossover sign in non-dysplastic hips was significantly associated with the development of arthritis.

**Conclusions:**

The prevalence of the crossover sign in hips in a Japanese population is similar to that reported in Western populations, despite the fact that FAI is believed to be less prevalent in the Japanese population. Furthermore, the presence of the crossover sign in non-dysplastic hip joints is associated with the development of arthritis. Based on our results, pincer-type impingement could be commonly associated with the development of arthritis in Japanese populations.

## Background

Femoroacetabular impingement (FAI) is one factor known to cause pain and osteoarthritis (OA) of the hips. First described by Ganz et al. in 2003 [1,2], FAI was identified in cases previously considered ‘primary hip OA’, in which initial radiography assessment suggested no hip joint abnormalities [3]. Generally, in the diagnosis of FAI, disorders such as hip dysplasia, rheumatoid arthritis, avascular necrosis of the femoral head, and fractures around the hip joint must be excluded. FAI may be divided into two subcategories based on the pathogenic mechanism: pincer type (impingement due to acetabular retroversion or acetabular overcoverage) or cam type (impingement due to an aspherical femoral head) [4]. Currently, several radiographic indicators have been described for the assessment of FAI [5-8].

While secondary OA due to hip dysplasia is common among Japanese populations, primary OA is seldom observed [9-11]. Because FAI is thought to be uncommon in Japan, relatively few epidemiological studies have addressed this issue. In their study of 817 Japanese patients who underwent surgery for OA of the hip, Takeyama et al. [12] reported that only 1.8% (17 of 946 hips) of hips examined demonstrated primary OA, with radiographic indicators of FAI present in only 0.6% of hips (6 of 946 hips), leading the authors to conclude that FAI is indeed rare in Japanese populations. However, since their study examined only surgically treated osteoarthritic hips, including those with severe OA, the true prevalence of FAI in the Japanese population remains to be determined.

Degenerative changes in the hip are influenced by the severity of hip dysplasia [13,14]. Gosvig et al. [15] examined 4,151 individuals from the Copenhagen Osteoarthritis Substudy cohort between 1991 and 1994 for radiographical malformation and investigated the estimated risk of osteoarthritis between the hip morphological malformation. They concluded that a deep socket and a pistol grip deformity were common radiographic findings and associated with an increased risk of hip OA. However, features of hip morphology among Europeans are thought to differ slightly from those of Asian populations, and whether their conclusions can be adapted to a Japanese population is unclear.

Here, to determine the prevalence of radiographic indicators of FAI in the hips of Japanese patients, we investigated the hips of patients who reported no complaints. We also determined whether or not these indicators represent risk factors for degeneration of the hip joint by performing observational radiographic assessment.

### Patients and methods

This study was designed as a retrospective observational cohort study to assess the prevalence of radiographic indicators of FAI and how these indicators affected the natural history of the hip joint in patients without a complaint of OA. Approval from the Institutional Review Board Committee was obtained for this study.

### Patients

We retrospectively examined the records of 411 Japanese patients ≥20 years of age who underwent unilateral hip osteotomy at our institution between 1971 and 2003. Of the 411 eligible patients, we identified 103 cases with a diagnosis of category A hip disease according to the Charnley classification [16] with no radiological evidence of arthritic changes on the non-operated side at the time of surgery (i.e. abnormal clinical findings on the operated side only). Patients were excluded based on a history of inflammatory arthritis, osteonecrosis of the femoral head, significant trauma, and significant malformation of the femoral head (e.g. slipped capital femoral epiphysis or Legg-Calve-Perthes disease). This study cohort offered the opportunity for long-term follow-up at a single institution, allowing the observation of the natural history of the hip in the absence of physical complaints. Furthermore, radiographs of the contralateral side were collected under the same conditions annually after the operation. Patients who underwent hip osteotomy were selected because they tended to have a relatively long follow-up period and to be relatively younger than those who underwent total hip arthroplasty (THA). These cases were further reviewed and excluded from analysis if radiographs taken at the time of surgery were unavailable, if radiographs in the anteroposterior (AP) view were considered inappropriate for evaluation according to the criteria proposed by Siebenrock et al. [17], or if the patient had a follow-up period of less than 2 years. A total of 87 patients were ultimately eligible for analysis, comprising 7 males and 80 females with a mean age of 44.2 years (range 20–56 years) at the time of surgery.

### Radiographic assessment

The centre-edge (CE) angle of the non-operated hip joint was measured in all 87 patients. Hip joints with a CE angle ≤20° were defined as dysplastic, while those with a CE angle >20° were defined as non-dysplastic. The AP plain radiographs of the hip joint were assessed for the presence of pistol grip deformity, which indicates cam-type FAI. We defined a pistol grip deformity as prominent lateral offset of the femoral head-neck junction by drawing a circle around the femoral head on the AP view. In addition, we assessed the crossover sign, an indicator of acetabular retroversion, which, in turn, is indicative of pincer-type FAI [18]. The presence of arthritic changes on AP plain radiographs was evaluated at the latest follow-up radiograph available for each patient using the Tönnis classification, and a grade ≥2 was defined as arthritic change [19]. If the patients had any further operations in addition to the investigation of the current study, we defined the time of surgery as the follow-up endpoint.

All radiological assessments were performed by a single observer (KF). The radiographs were reviewed again at least 6 months after the initial review by the same observer. Consequently, we defined cases as positive for dysplasia, pistol grip deformity, crossover sign, or arthritic change when both assessments were in agreement. The mean duration of the evaluation period was 139 months (range 56–479 months).

### Statistical analysis

Continuous variables are presented as median values with the range in parentheses, while categorical variables are given as *N* (%); assessment was conducted using Pearson's chi-squared or Fisher's exact test, as appropriate. An endpoint was defined as the presence of arthritic change and/or hip surgery. Kaplan-Meier analysis and Cox proportional hazard regression were used to evaluate the prognosis of different groups. For the Kaplan-Meier estimate of survival curves, we truncated the data at 180 months of follow-up to maintain a sufficiently high number at risk. Patients with a survival time greater than 180 months were recorded as 180 months, and events that occurred after the end of the 180-month follow-up period were computed as censored data. All reported *P* values are two-sided, and values of 0.05 or less were regarded as statistically significant. Analyses were performed using SPSS version 17.0 software (SPSS Inc., Chicago, IL, USA).

## Results

### Prevalence of radiographic femoroacetabular impingement

Among the 87 hips assessed in our study, 38 hips (43.6%) were dysplastic and 49 hips (56.4%) were non-dysplastic. The mean (range) CE angle was 16.8° (5°–20°) in the dysplasia group and 28.1° (21°–43°) in the non-dysplasia group. The mean age of subjects did not statistically differ between the two groups. Although the total number of males was very small, the number of males in the dysplasia group was significantly larger than that in the non-dysplasia group (*P* = 0.019). The CE angle was significantly smaller in the dysplasia group (*P* < 0.0001). No pistol grip deformity was observed, but crossover sign (as a radiographic indicator of pincer-type impingement secondary to acetabular retroversion) was identified in 9 of the 38 hips (23.7%) in the hip dysplasia group and 15 of the 49 hips (30.6%) in the non-dysplasia group (Table 1). Although the prevalence of the crossover sign tended to be higher in the non-dysplasia group, the difference was not statistically significant (*P* = 0.47).

**Table 1:** Background and radiographic findings in the subjects

	**Dysplastic hips (*n*** **= 38)**	**Non-dysplastic hips (*n*** **= 49)**
Mean age (years)	41.29 (20–56)	46.38 (21–56)
M/F (*n*)	6:32	1:48
Follow-up term (months)	123 (56–365)	152 (60–479)
CE angle (deg)	16.8 (5–20)	28.1 (21–43)
Pistol grip deformity (*n*)	0	0
Crossover sign (*n*)	9	15

### Influence of acetabular retroversion on the development of arthritis

**Figure 1:**
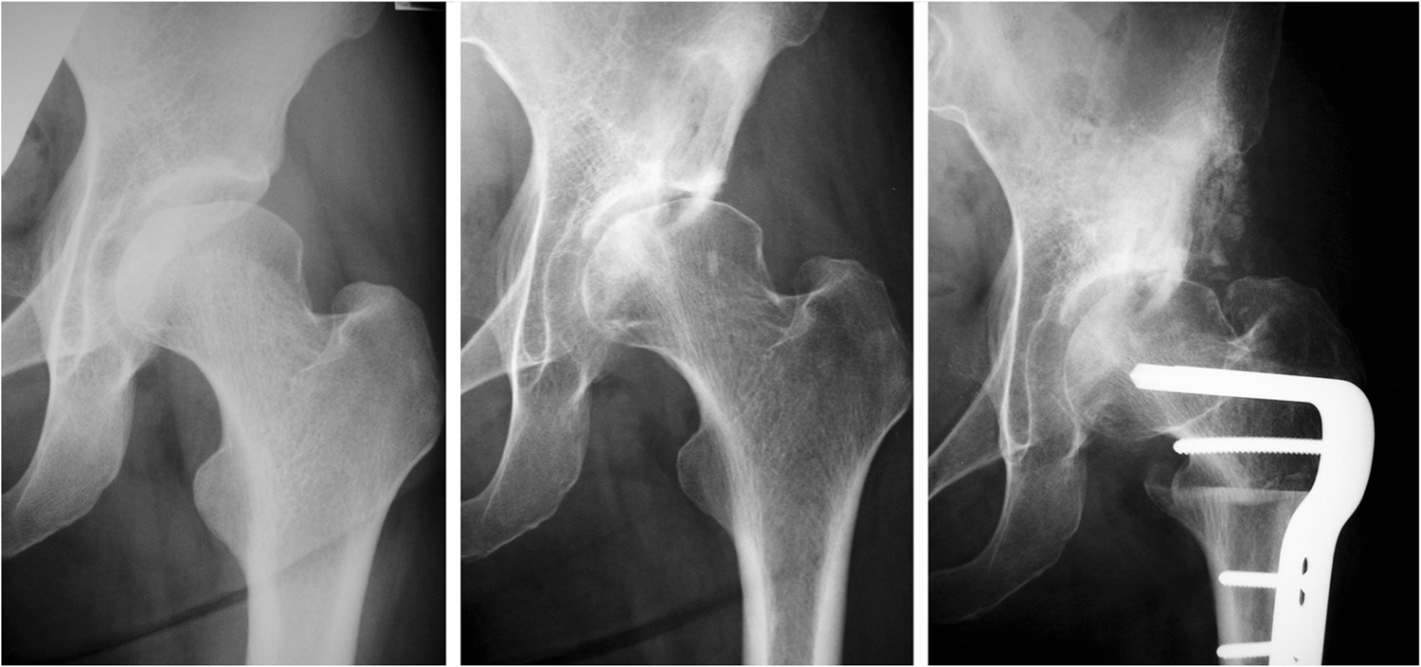
**Case that developed arthritic changes (non-dysplastic, positive crossover sign). ***Left*: radiograph before the follow-up (age 34 years old, female); the patient underwent Chiari osteotomy on the contralateral side for developmental dysplasia. *Middle*: radiograph from 16 years after the beginning of the follow-up (age 50 years old); the joint space was slightly narrower, and a bone cyst was noted on the acetabular side. *Right*: radiograph after the end of the follow-up (age 51 years old); the patient complained of severe pain in the hip. Therefore, she underwent femoral subtrochanteric varus osteotomy and allogenic bone grafting into the bone cyst to prevent collapse.

The mean (range) follow-up time of the entire study population was 139 (56–479) months, comprising 123 (56–365) months for the dysplasia group and 152 (60–479) months for the non-dysplasia group. The follow-up time did not significantly differ between the two groups (*P* = 0.11). In the dysplasia group, a total of 13 of 38 hips (34.2%) were classified as Tönnis grade 2 or higher, including 1 hip that underwent rotational acetabular osteotomy (RAO) and 5 hips that underwent THA. Of the 13 dysplastic hips with arthritic changes, 3 (23.6%) demonstrated a crossover sign on radiography, indicating the presence of acetabular retroversion. In the non-dysplasia group, 11 of 49 non-dysplastic hips (22.4%) had arthritic changes, including cases that required RAO (1 hip), femoral subtrochanteric varus osteotomy (1 hip), or THA (2 hips). Of the 11 non-dysplastic hips with arthritic changes, a crossover sign was observed in 8 hips (72.7%) (Figure 1).

**Figure 2:**
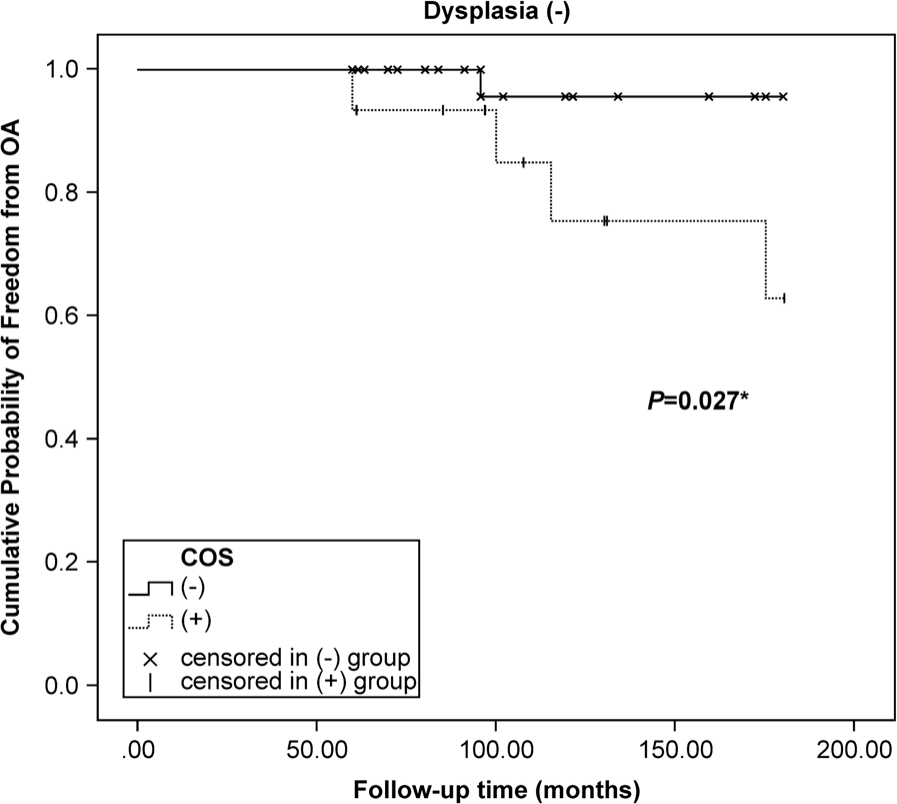
**Influence of acetabular retroversion on the development of arthritis in non-dysplastic hips.** After a follow-up period of up to 180 months, the Kaplan-Meier estimate showed that patients with non-dysplastic hips and the crossover sign (*COS*) had an inferior prognosis (*P* = 0.027, log-rank test).

**Figure 3:**
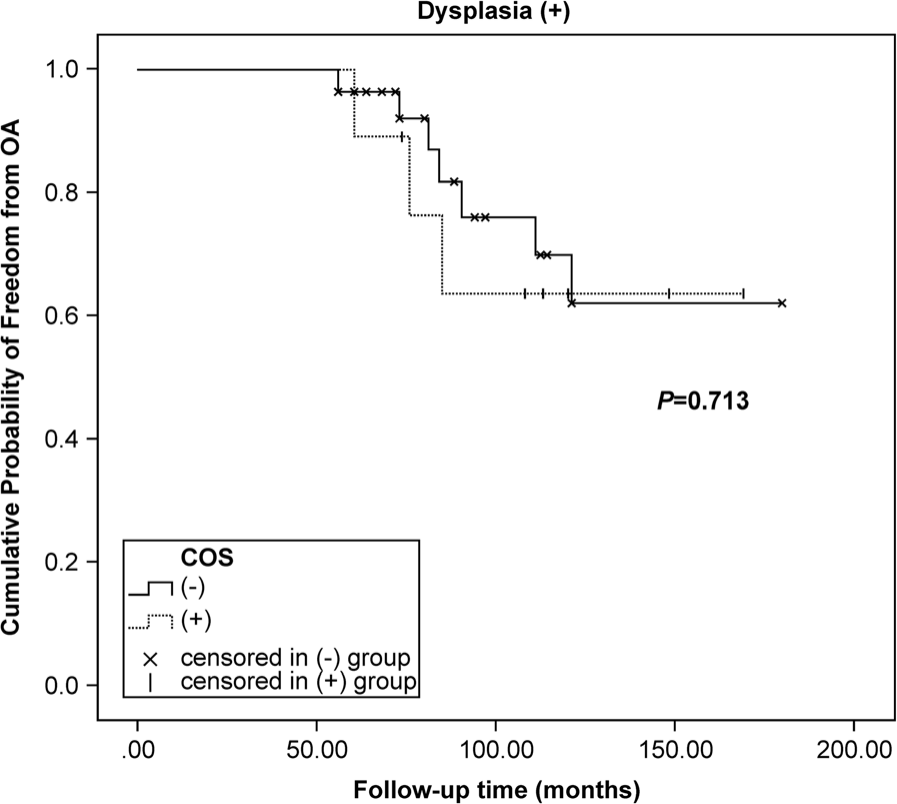
**Influence of acetabular retroversion on the development of arthritis in dysplastic hips.** After a follow-up period of up to 180 months, the Kaplan-Meier estimate showed that the presence of the crossover sign (*COS*) did not affect the prognosis of patients with dysplastic hips (*P* = 0.713, log-rank).

Survival analysis was used to evaluate whether the presence of the crossover sign was associated with the development of hip arthritis. Dysplasia was defined as an interaction factor of the crossover sign (*P* = 0.024). After a follow-up period of up to 180 months, the Kaplan-Meier estimate revealed that patients with non-dysplastic hips and the crossover sign had an inferior prognosis (*P* = 0.027, log-rank, Figure 2), but prognosis was not significantly affected in the dysplasia group (*P* = 0.713, Figure 3). The hazard ratio (95% confidence interval [CI]) of the crossover sign and arthritis was 9.45 (1.99–44.91) for individuals with hip dysplasia (*P* = 0.005) and 0.98 (0.25–3.81) for those without hip dysplasia (*P* = 0.98), after a total follow-up period of 479 months. These findings were independent of age.

## Discussion

In the current study, we radiographically examined the prevalence of femoroacetabular impingement (FAI) in non-operated, asymptomatic, non-osteoarthritic hip joints in Japanese patients and assessed the development of arthritis in those with FAI. Since Ganz et al. first described FAI in 2003 [1], the concept has attracted increasing attention, particularly in Europe and the USA.

The prevalence of FAI in Asian countries has been reported by Takeyama et al. [12], and this study suggested that FAI is uncommon among the Japanese population. In the current study of a Japanese population, the pistol grip deformity was absent, a finding consistent with the report of Takeyama et al. [12], while the prevalence of the crossover sign was much higher than in their investigation. Furthermore, in the current study, survival analysis revealed that the presence of the crossover sign in non-dysplastic hips was significantly associated with the development of arthritis. The difference in the prevalence of FAI between the current study and that of Takeyama et al. [12] may be due to differences in study population characteristics: we examined non-operated hip joints with no clinical or radiological evidence of arthritis (category A according to Charnley classification), while Takeyama et al. [12] examined hip joints that underwent surgery for moderate to severe OA. Furthermore, retrospective detection of radiological evidence of FAI in moderate to severe hip OA may have been difficult in the earlier study, while we experienced relative ease of detection of FAI in non-arthritic hip joints.

The current study population was not equally distributed according to sex (i.e. 80 females and 7 males among 87 subjects). Jingushi et al. reported a nationwide epidemiologic study regarding hip OA in the Japanese population, in which they examined 720 hips from 15 institutions and reported that the proportion of females to males was 90% to 10% [20]. The percentage of females was similar in the current study. Therefore, the sex distribution of subjects in the present study appears to accurately reflect that of Japanese patients who have this hip disorder.

In the current study of a Japanese population, the crossover sign (as an indicator of acetabular retroversion) was present in 30.6% of hips without dysplasia, which is comparable to the prevalence reported by Jamali et al. [21] (33%) in a cadaveric study, suggesting that the prevalence of acetabular retroversion may be similar in Japanese and Western populations. The observed prevalence of acetabular retroversion in dysplastic hips was 23.6% in the present study, whereas studies by Ezoe et al. [22] and Fujii et al. [23] found an identical rate of 18% among Japanese populations. Fujii et al. [23] also noted that pain onset was significantly earlier in dysplastic hips with acetabular retroversion compared to those without acetabular retroversion, suggesting that acetabular retroversion was pathologically significant in dysplastic hips. Whether this concept applies to the pincer type of FAI is unknown. In the aforementioned study, the authors stated that acetabular retroversion in hip dysplasia resulted from deficient posterior coverage of the acetabulum, raising the possibility that acetabular retroversion in dysplastic hips may have different pathomorphological significance and mechanical characteristics from the original concept of pincer-type FAI.

Interestingly, the presence of the crossover sign correlated with the development of arthritis in non-dysplastic hips. Although Siebenrock et al. [24] previously described symptomatic anterior impingement in the setting of acetabular retroversion, they did not discuss the association between acetabular retroversion and the development of arthritic changes. By contrast, the crossover sign was not associated with the development of arthritic changes in dysplastic hips in the present study, suggesting that the crossover sign may only be predictive of hip arthritis in non-dysplastic hips. Tanzer and Noiseux [25] reported that 62.5% of hips (125 of 200) with idiopathic OA undergoing THA demonstrated pistol grip deformity and no acetabular morphological abnormality, suggesting that anterior impingement was a cause of arthritis. Other reports have also suggested that FAI is associated with the development of arthritis [26,27]. Since many of these studies were cross-sectional and not cohort observational studies, the risk of development of arthritis in hips with FAI remains unknown.

The natural history of 43 hips with a morphological abnormality that may reflect FAI and mild to moderate arthritis was documented by Bardakos and Villar [28] with a long-term follow-up of more than 10 years (mean of 127.1 months). Progression of arthritis was noted in 28 of 43 hips and was associated with a significantly lower median proximal femoral angle and posterior wall sign. In addition, the crossover sign was not significantly associated with progression of arthritis in their study, and while a difference was seen between the two groups, it was not statistically significant (*P* = 0.08). The size of the current study (*n* = 87) was larger than that of the previous report, and the follow-up time was equivalent. In the current study, the crossover sign was significantly associated with progression of arthritis in non-dysplastic hips. These previous findings may differ from those of the present study because Bardakos and Villar [28] examined the progression of existing arthritis (i.e. patients already suffered from mild to moderate OA, Tönnis grade 1 or 2), in contrast to our present study, in which we examined the development of OA in previously OA-free hip joints. Further cohort observational studies are needed to fully clarify this point.

Several limitations to the current study warrant mention. Most importantly, the study size was relatively small, and the follow-up term was too short (mean duration of the evaluation period of 139 months) to draw any definitive conclusions as an observational study. Accordingly, the number of subjects reaching clinical endpoints is also small (e.g. only three dysplastic hips with a positive crossover sign developed arthritic changes). Confidence intervals, therefore, were too wide. Further studies are needed to clarify the natural history of non-OA hips in the Asian population and the estimated risk of OA in hips demonstrating morphological malformation.

Second, we selected study subjects from among patients who underwent unilateral hip osteotomy for their hip disorder. Genetic influences have recently been implicated in the aetiology of FAI [29]. Hartofilakidis et al. examined the long-term outcome of 96 asymptomatic hips with radiological evidence of FAI, as in the current study design [30]. They found that the presence of idiopathic osteoarthritis of the contralateral diseased hip was predictive of the development of osteoarthritis on the asymptomatic side. We could not clarify in the current study whether the influence of genetic background or contralateral disease affected the natural history of our subjects because we did not have a control group.

In addition, lateral radiographs were not analysed, as was recommended by Tannast et al. [8], because our institute's routine examination of hip joint radiographs involved both AP and frog-leg lateral views, and some patients were examined in the AP view only. Therefore, we defined the pistol grip deformity as an indicator of cam-type FAI. The need to examine lateral images was suggested in a study by Gosvig et al. [31], which reported low detection rates of pistol grip deformity (3.2% in males, 5.4% in females) on examination of AP radiographs alone. While pistol grip deformity was absent in the current study, we cannot conclude that the prevalence of cam-type FAI in Japanese patients is lower than that in Western populations, as cam-type impingements may have been under-detected in our population due to our failure to observe decreases in the anterior offset or the presence of osseous bumps located strictly anteriorly at the femoral neck, which were not identified as the pistol grip deformity.

Overall, the results of our study confirm that the prevalence of acetabular retroversion may be similar in Japanese and Western populations. Furthermore, acetabular retroversion might be a predictor of the development of arthritic change in the Japanese population. We suggest that doctors, particularly in Asia, pay more attention to this radiographic abnormality that may reflect FAI.

## Conclusions

In the current study, the prevalence of the crossover sign in Japanese natural hip joints was found to be comparable to that previously reported in Western populations. Furthermore, the presence of acetabular retroversion was associated with the development of hip arthritis in non-dysplastic but not in dysplastic hip joints. Based on our results, pincer-type impingement could be commonly associated with the development of arthritis in Japanese populations.

## Abbreviations

AP: anteroposterior; FAI: femoroacetabular impingement; OA: osteoarthritis; RAO: rotational acetabular osteotomy; THA: total hip arthroplasty.

## Competing interests

The authors declare that they have no competing interests.

## Authors’ contributions

KF and KU designed the study and collected and performed the analysis of the data. KF wrote the manuscript. NT, MM and TY participated in the data collection. MI and MT supervised the study. All authors read and approved the final manuscript.
